# The Co-Occurrence of Temporomandibular Disorders in Patients Diagnosed with Tinnitus: A Systematic Review with Meta-Analysis

**DOI:** 10.3390/jcm14061836

**Published:** 2025-03-08

**Authors:** Michał Bury, Kacper Nijakowski, Anna Majewska, Jakub Jankowski, Anna Surdacka, Dorota Hojan-Jezierska

**Affiliations:** 1 Department of Hearing Healthcare Profession, Chair of Biophysics, Poznan University of Medical Sciences, 60-780 Poznan, Poland; amajewska@ump.edu.pl (A.M.); djeziers@ump.edu.pl (D.H.-J.); 2 Department of Conservative Dentistry and Endodontics, Poznan University of Medical Sciences, 60-812 Poznan, Poland; jjankowski41@wp.pl (J.J.); annasurd@ump.edu.pl (A.S.)

**Keywords:** tinnitus, temporomandibular disorder, temporomandibular joint

## Abstract

**Background/Objectives:** Persistent and distressing tinnitus and TMDs may significantly impair the quality of patients’ lives. Problems are particularly severe for those who experience both. Although the exact causes of this association are not fully understood, several hypotheses connect TMD conditions with otological symptoms. **Methods:** The systematic review presented involved records published between 1 January 2004 and 27 November 2024, in the databases PubMed, Scopus, and Web of Science, according to the PRISMA guidelines. The search queries included “temporomandibular disorder” and “tinnitus”. Inclusion and exclusion criteria were predefined according to PECOS. A risk of bias assessment and meta-analyses were performed. The study’s protocol was registered in PROSPERO (CRD42024533293). **Results:** Eleven studies included in this review fulfilled all the predefined criteria. In a sample of 114,071 tinnitus patients, 50.99% (95% CI: 33.31–68.54) also had coexisting TMDs. Furthermore, tinnitus patients had more than 2.2 times higher odds of having TMDs compared to the control group (*p* = 0.002 for random effects). The absence of data regarding randomisation, blinding, and sample size justification was the most frequent risk of bias. **Conclusions:** Tinnitus frequently co-occurs in patients who suffer from TMDs. It is essential to expand the diagnostic evaluation of patients to confirm its presence and assess its impact on their quality of life. Additionally, dental consultation should be considered to evaluate patients about TMD signs and symptoms when suffering from tinnitus.

## 1. Introduction

Tinnitus is used to describe the continuous sensation of sounds inside the head or ear without any external acoustic stimulus in the auditory pathway [1]. It accompanies a variety of diseases affecting the auditory organ, and may coexist with some systemic or organ-associated conditions [2]. The perceived sound may be experienced centrally in the head, binaurally or monoaurally, and may present with various frequencies and intensities [3]. Patients identify the sensation of tinnitus as a ringing, buzzing, or whistling sound. Pathophysiological models assume that tinnitus may stem from the brain’s inability to adapt to the fact that sensory information is not delivered from the inner ear—a key element in the aetiology of its onset. This, in turn, leads to the activation of a compensatory mechanism in the brain, which results in increased neuronal activity along the auditory four-neuron pathway. This heightened activity is thought to be a maladaptive response, where the brain “fills in the gaps” with spontaneous neural firing, which patients perceive as tinnitus. Essentially, tinnitus may arise because the brain attempts to adjust to missing auditory input but ends up generating abnormal neural signals instead [4].

In 1995, Jastreboff performed the precise classification of tinnitus according to its nature, and differentiated it into subjective and objective [5]. In terms of epidemiology, subjective tinnitus is the most commonly observed form, and it derives exclusively from mechanical activity within the inner ear and impaired function of the central nervous system or of the vestibulocochlear nerve (CN VIII). The underlying causes of subjective tinnitus are most frequently otologic in character [2,6]. In contrast, objective tinnitus, also referred to as somatosound or pseudo-tinnitus, is a type of noise that is perceptible not only for the patient, but also for other individuals. In fact, objective tinnitus may be detected and recorded using measuring devices. Its underlying causes include cardiovascular disorders, intracranial hypertension, soft palate myoclonus, and myoclonic spasms of the middle ear muscles, and it may also be associated with temporomandibular joint disorders (TMDs). In the case of the latter, tinnitus has been reported in approximately 65% of patients [7]. Interestingly, the literature suggests that mandibular crowding may also potentially contribute to the occurrence of tinnitus [8,9].

Various experimental studies have emphasised that tinnitus is a diversified phenomenon and that it cannot be regarded as a disease, but rather as a subjective symptom. Conversely, temporomandibular disorders involve the complex musculoskeletal system of the jaw as well as the surrounding structures. Therefore, tinnitus and TMDs constitute two complex medical issues, which may interact in a number of respects [10]. Crucially, the impact of craniofacial health issues on the severity of tinnitus remains poorly understood. The pathophysiology of TMDs may be linked to otological disorders, due to the anatomical proximity of the cochlea and temporomandibular joint (TMJ), through the connections of the nerve and blood vessels. Furthermore, temporomandibular joint inflammation or mechanical damage may result in pressure on nerves and blood vessels, which in turn may contribute to a range of ear symptoms [11]. These involve a feeling of ear fullness (74.9%), ear pain (65.0%), increased otalgia (55.1%), tinnitus (52.2%), vertigo (40.9%), and dizziness (21.9%), as well as hearing loss (39.0%) [7].

TMDs have a multifactorial aetiology, involving both objective (biological) and subjective (psychosocial) factors. The development of TMDs may result from a combination of genetic predisposition, the mechanical overload of the masticatory muscles (e.g., excessive parafunctional activity such as bruxism), structural changes within TMJ (e.g., disc displacement, osteoarthritic changes), or trauma. Additionally, inflammatory processes and systemic conditions, such as rheumatoid arthritis, can contribute to TMDs. Psychosocial factors, including stress, anxiety, and pain-related central sensitisation, also play a significant role in the onset and persistence of symptoms [12,13]. TMDs are observed in approximately 34% of the population [14]. Hence, TMDs, which are quite common, may significantly affect the severity of tinnitus. Additionally, research results indicate that up to 57.5% of dental patients may experience such issues simultaneously [15]. Therefore, it is vital to approach TMD patients holistically, also taking into consideration the potential otological and audiological complaints [16]. Although, clinically, tinnitus is not a life-threatening condition, it significantly decreases the quality of patients’ lives; thus, the diagnosis necessitates the interdisciplinary involvement of multiple specialists, particularly in the areas of audiology, neurology, dentistry, and psychiatry [17].

TMJ is an integral part of the masticatory system; however, TMDs encompass a broader range of conditions, affecting not only the TMJ but also the associated muscles and structures. According to the Diagnostic Criteria for Temporomandibular Disorders (DC/TMDs), the diagnostic process should include a thorough anamnesis, validated patient questionnaires, and a detailed clinical examination [18]. Clinical assessment involves the palpation of the masticatory muscles for tenderness, the evaluation of mandibular mobility, and the assessment of any associated symptoms, including pain and joint sounds. Imaging techniques, such as X-rays, computed tomography (CT), ultrasound (US), or magnetic resonance imaging (MRI), should only be used when necessary to confirm the diagnosis or to guide treatment decisions [19,20,21]. The diagnostic process of TMDs involves the careful analysis of information derived from the patient’s subjective and objective examinations, which should subsequently lead to the identification of a specific disorder. In cases where a patient presents with a single disorder, the diagnosis is a relatively routine procedure. However, the clinician needs to take into consideration the fact that the patient may simultaneously suffer from more than one disorder, involving more than one structure, and that these may interact with each other [22].

When patients report otological symptoms, audiological diagnostics should be considered in order to assess the functioning of the auditory pathway, from the peripheral organ to the auditory centres located in the central nervous system. Moreover, neurological, cardiological, and psychiatric consultations are also recommended. Our systematic review aimed to evaluate the co-occurrence of TMDs in patients diagnosed with tinnitus.

## 2. Materials and Methods

### 2.1. Search Strategy and Data Extraction

The systematic review presented was conducted based on the records published between 1 January 2004 and 27 November 2024, according to the guidelines of the Preferred Reporting Items for Systematic Reviews and Meta-Analyses (PRISMA) [23], and involved the databases PubMed, Scopus and Web of Science. The search queries were as follows:

- For PubMed: (“temporomandibular disorder” [MeSH Terms] AND (“tinnitus”);
- For Scopus: TITLE-ABS-KEY (“temporomandibular disorder” AND “tinnitus”);
- For Web of Science: TS = (“temporomandibular disorder” AND “tinnitus”).

Two independent researchers reviewed the records for the title, abstract, and full text. The studies included in this review fulfilled all the predefined criteria according to PECOS (“Population”, “Exposure”, “Comparison”, “Outcomes”, and “Study design”), as shown in Table 1. A detailed search flowchart is included in the Section 3. The study protocol was registered in the international prospective register of systematic reviews PROSPERO (CRD42024533293).

The results of the meta-analysis were presented as forest plots using MedCalc Statistical Software, version 22.014 (MedCalc Software Ltd., Ostend, Belgium). The pooled prevalence and odds ratios of temporomandibular disorders in patients with tinnitus were calculated. Egger’s test was used to assess potential publication bias in a meta-analysis.

### 2.2. Quality Assessment and Critical Appraisal for the Systematic Review of Included Studies

The risk of bias in each individual study was assessed according to the “Study Quality Assessment Tool” issued by the National Heart, Lung, and Blood Institute within the National Institute of Health [24]. Two independent researchers completed the questionnaires, and any disagreements were resolved through discussion.

The level of evidence was assessed using the classification of the Oxford Centre for Evidence-Based Medicine levels for diagnosis [25].

## 3. Results

**Figure 1:**
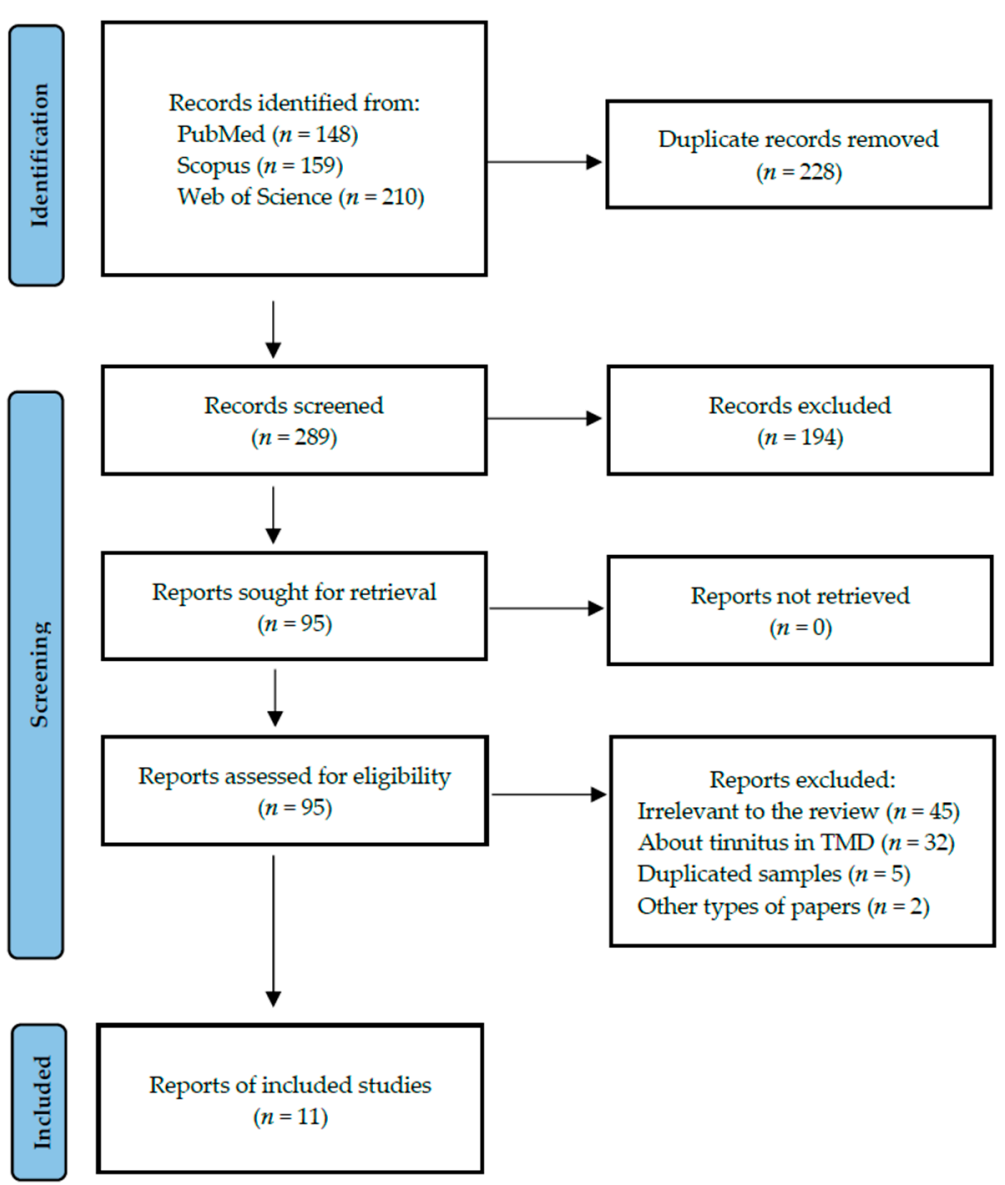
PRISMA flow diagram presenting search strategy.

A total of 11 studies fulfilled the search criteria and were subsequently included in our critical review. The analysed papers provided data collected in six different countries. Figure 1 shows a detailed selection strategy of the records. The inclusion and exclusion criteria are presented in Table 1.

Table 2 provides the details of the included studies, such as authors and year of publication, setting, study design, involved participants, tinnitus diagnosis, and clinical criteria for TMDs.

Our meta-analyses comprised all 11 studies (8 cross-sectional and 3 case-control studies) that reported TMD prevalence in patients suffering from tinnitus.

**Figure 2:**
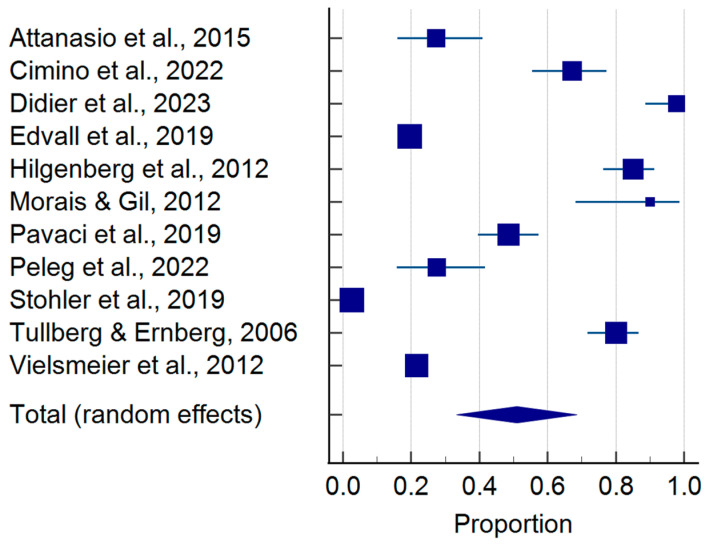
Pooled prevalence of temporomandibular disorders in tinnitus patients (squares—proportions for individual studies; diamond—polled proportion) [1,3,16,26,27,28,29,30,31,32,33].

**Figure 3:**
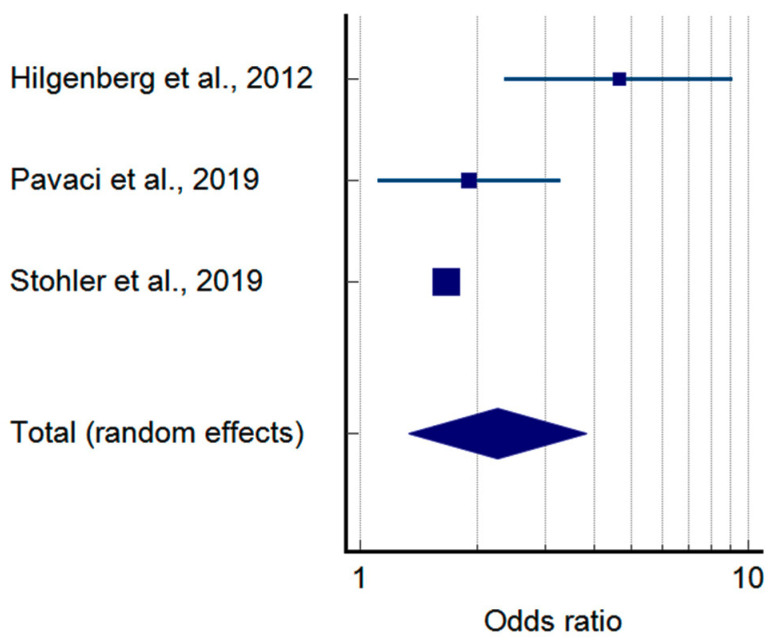
Pooled odds ratio of temporomandibular disorders in tinnitus patients based on case-control studies (squares—odds ratios for individual studies; diamond—polled odds ratio) [29,31,32].

The pooled prevalence of TMDs in tinnitus patients is shown in Figure 2. According to the sample of 114,071 tinnitus patients, 50.99% (95%CI: 33.31–68.54) presented with co-occurring TMDs (Table 3). Moreover, considering the case-control approach, the odds of developing a TMD were more than 2.2 times higher in tinnitus patients as compared to the controls (*p*-value = 0.002 for random effects)—Figure 3 and Table 4.

**Figure 4:**
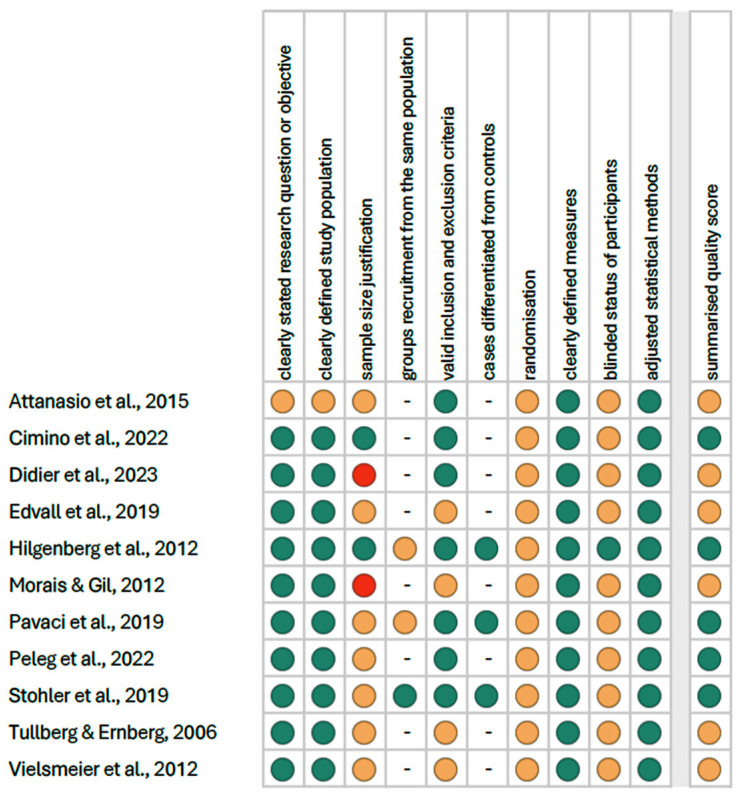
Quality assessment, including the main potential risk of bias (risk level: green—low, yellow—unspecified, red—high; quality score: green—good, yellow—intermediate, red—poor) [1,3,16,26,27,28,29,30,31,32,33].

The summarised quality assessment is illustrated in Figure 4. The most common sources of bias were the lack of information on randomisation (in all studies), blinding (in 10 studies), and justification for sample size (in 9 studies). The critical appraisal was conducted by assigning points to each criterion based on potential risk (scoring: 1—low risk; 0.5—unspecified; 0—high risk). According to our analysis, 5 studies (45.5%) were classified as having “good” quality (≥80% of the total score), while 6 studies (54.5%) were categorized as having “intermediate” quality (≥60% of the total score).

All studies included corresponded to the third or fourth level of evidence (case-control and cross-sectional studies) as defined by the Oxford Centre for Evidence-Based Medicine’s classification for diagnostic research [25].

## 4. Discussion

Tinnitus is the sensation of sound that occurs despite the absence of an external acoustic stimulus, and it is more frequently subjective than objective in nature. According to the literature review conducted, tinnitus may also accompany temporomandibular disorders (TMDs), which affect the complex musculoskeletal system of the jaw, as well as the surrounding structures. Although tinnitus and TMDs are two separate and complex medical issues, they frequently coincide. Although the exact causes of the association are not fully understood, several hypotheses can be put forward. The coexistence of TMDs and tinnitus may result from the proximity of the middle ear and the temporomandibular joint (TMJ) [34,35,36]. This close anatomical and functional relationship between the two structures is facilitated by the anterior malleolar ligament (AML), the sphenomandibular ligament (SML), and the discomalleolar ligament (DML). Furthermore, unlike the AML, the DML plays a pivotal role in the mobility of the malleus, the structure which contributes to TMJ function and plays a role in cases of forward displacement of the intervertebral disc. The above-mentioned ligaments extend during the TMJ movement, thereby affecting middle ear structures, such as the eardrum and malleus [37]. The relationship between TMJ and the middle ear depends on the performance of specific ligaments, their structure (collagenous or elastic), and the width of the petrotympanic fissure. In addition, the overactivity of the masseter muscles may be associated with the development of tinnitus and TMDs, since these structures, connected to the TMDs, share trigeminal innervation with the muscles involved in the function of the Eustachian tube, including the lateral and medial pterygoid muscle [38]. Interestingly, disturbed pressure regulation in the Eustachian tube may constitute a significant factor in increasing the risk of tinnitus [38].

This association is further corroborated by the somatosensory theory, whereby chronic pain or psychosocial disorders, such as depression or anxiety, trigger changes in the central nervous system, resulting in sensory hypersensitivity and an altered perception of auditory stimuli [36,39,40]. Moreover, recent studies also suggested that this connection could stem from increased intestinal permeability, disrupted gut microbiota, and abnormal metabolism of tryptophan, i.e., a biomarker of chronic musculoskeletal pain. It is of note that tryptophan is a precursor of serotonin and melatonin, which are critical in the activation of microglia (e.g., in the ES gastrointestinal tract) and participate in the regulation of chronic orofacial pain. The impaired metabolism of tryptophan may lead to increased pain sensitivity and depression which, in turn, affect both TMDs and tinnitus [41]. In fact, other studies conducted reported a link between depression, TMDs, and the development of tinnitus. Furthermore, researchers also suggest that another factor, i.e., TMJ herniation, may also potentially trigger tinnitus, although the disorder itself is rare [42]. The above-mentioned observations are in line with the findings of our meta-analysis, according to which more than 50% of patients with tinnitus also presented with TMDs, and show that there is a correlation between these disorders. Additionally, the relation between the two has also been pointed out by several other researchers in their reports.

An epidemiological study by Hilgenberg et al., investigating the coexistence of tinnitus and TMDs, found a strong correlation between the two disorders [29]. Other researchers found that 30.6% of dental patients suffering from TMDs also showed a history of tinnitus [27], whereas 22% of their studied group, diagnosed with tinnitus, presented with TMDs [3]. Furthermore, the prevalence of TMDs in tinnitus patients was 85%, which is consistent with the findings of similar studies. Additionally, they also observed that tinnitus was more common in women than in men. In terms of women, 54% of patients suffered from tinnitus with TMDs, while 33% presented with tinnitus without TMDs. Conversely, 46% of male participants experienced tinnitus with TMDs, whereas 67% developed tinnitus without TMDs [3].

Notably, the severity of tinnitus was affected by bruxism, and chronic facial pain was greater in patients with tinnitus and sleep bruxism. Tinnitus was more common when it co-occurred with painful TMDs; however, no correlation was found between somatosensory tinnitus and the development of TMDs. The results obtained by Edvall et al. [28] indicated that only 19% of individuals with existing tinnitus reported temporomandibular joint issues [28]. Conversely, a study by Peleg et al. [16] showed significant statistical differences between the presence of tinnitus and bilateral TMDs presenting with bruxism.

In terms of epidemiology, one of the studies found that the proportion of patients diagnosed simultaneously with TMDs and tinnitus was 85%. Bilateral tinnitus was observed in 47% of subjects, unilateral tinnitus was observed in 43%, and central tinnitus was observed in only 10% [27]. Another literature review showed that, in 79% of cases, tinnitus was reported on the right side, while it was reported on the left in 85% of cases, and 63% of individuals experienced bilateral tinnitus [33]. In turn, in a combined analysis of the tinnitus prevalence, a difference was reported in 24 women and 27 men. In the majority of cases, tinnitus was found bilaterally in both ears and was observed in 72.5% of cases [33].

In addition to tinnitus, clinical diagnosis reports the existence of a correlation between otological symptoms and TMD progression. In their study, Hildenberg et al. screened 785 patients for TMDs and excluded objective tinnitus. They showed that patients with TMDs and tinnitus presented with otalgia (27%), dizziness (52%), a sense of hearing loss (62%), and auditory hypersensitivity, combined with misophonia (26%) [29].

The study by Manfredini et al. [43] involved 238 patients with TMDs who were tested for tinnitus; the mean age of the subjects was 49.3 ± 13.5 years. The authors demonstrated that tinnitus was present in 30.4% of individuals with TMDs. This finding differs from the observations of the study by Silva et al. [44], in which tinnitus was present in 61.3% of TMD cases. However, it is of note that the Manfredini study group was approximately half the size of the Silva study group, and the mean age was also different. The high prevalence of tinnitus in the study of Silva may be due to the difference in the age of the participants, where the maximum age was 88 years. Age-related processes, senile hearing loss, comorbidities, and inflammation of the ear and vestibular nerve all contribute to the increased sensation of tinnitus. In the analysed group, TMD patients most frequently reported otological symptoms, such as fullness in the ears (59%), hyperacusis with misophonia (80%), and dizziness occurring 1–2 times per week (27%) [39,40].

Both our analysis and the findings of studies from different sources reported different values in terms of the co-occurrence between tinnitus and TMDs. However, all studies confirmed a correlation between the two disorders. The study confirms a strong association between tinnitus and TMDs, but it does not establish a direct causal relationship. The cross-sectional nature of most studies prevents an understanding of the progression of TMD-related tinnitus. The meta-analysis includes studies with different methodologies, diagnostic criteria, and sample sizes, which may introduce variability in results. Standardised diagnostic tools for both TMDs and tinnitus are necessary to achieve better comparability. Most studies included rely on self-reported tinnitus rather than objective audiological assessments, which may introduce subjectivity in the findings.

Future research should focus on tracking patients over time to clarify whether TMDs precede or worsen tinnitus, and to assess the effectiveness of various interventions. Studies evaluating the impact of TMD treatments (e.g., occlusal splints, physiotherapy, cognitive behavioural therapy) on tinnitus symptoms could help develop targeted therapeutic approaches. Also, since depression, anxiety, and stress play significant roles in both TMDs and tinnitus, interdisciplinary studies integrating psychology, neuroscience, and dentistry can provide deeper insights.

## 5. Conclusions

On the basis of the aforementioned studies and analyses, it appears that tinnitus frequently co-occurs in patients who suffer from TMDs. Since persistent and distressing tinnitus may significantly impair the quality of patients’ lives, it is essential to expand the diagnostic evaluation of this patient group so that it includes tinnitus. If tinnitus is observed in the patient’s history and the tests confirm the presence of such a symptom, questionnaires assessing the impact of tinnitus on the quality of life should be applied. In addition, when investigating the cause of tinnitus, a dental consultation should be incorporated into the diagnostic protocol in order to assess for TMDs.

It also seems advisable to develop a common standard for the diagnostic management of patients presenting with TMDs and tinnitus. This would allow for a quicker assessment of the causes of tinnitus and the application of causal therapies, aiming to improve the patients’ quality of life.

## Figures and Tables

**Table 1:** Inclusion and exclusion criteria according to the PECOS.

Parameter	Inclusion Criteria	Exclusion Criteria
Population	patients with tinnitus—adults, both genders	patients with other neurological and hearing disorders
Exposure	temporomandibular disorders (TMDs)	other disorders of the oral cavity
Comparison	not applicable	
Outcomes	prevalence or odds ratio for TMDs in tinnitus patients	prevalence or odds ratio for tinnitus in TMD patients
Study design	case-control, cohort and cross-sectional studies	literature reviews, case reports, expert opinion, letters to the editor, conference reports
	published between 1 January 2004 and 27 November 2024	not published in English

**Table 2:** Detailed characteristics of included studies.

**Study**	**Setting**	**Study Design**	**Study Group (F/M, Age)**	**Control Group**	**Tinnitus Diagnosis**	**TMD Diagnosis**
Attanasio et al., 2015 [26]	Italy	cross-sectional	55 (NR), mean 41.8	n/a	chronic subjective tinnitus lasted at least for the last 12 months	Wilkes Classification Stages I and II
Cimino et al., 2022 [27]	Italy	cross-sectional	79 (37/42), 44.41 ± 11.7	n/a	episode of sound perception in the absence of external sources for more than 5 min at least 2 times a week	according to the Diagnostic Criteria for TMDs (DC/TMD) Axis I protocol
Didier et al., 2023 [1]	Italy	cross-sectional	47 (NR), NR	n/a	somatosensory tinnitus	according to the diagnostic criteria for TMD Axis I (DC/TMD), as any condition associated with pain and dysfunction of the masticatory muscles or TMJ
Edvall et al., 2019 [28]	Sweden	cross-sectional	2482 (1222/1260)	n/a	according to Tinnitus Sample Case History Questionnaire	self-reported TMJ problems
Hilgenberg et al., 2012 [29]	Brazil	case-control	100 (84/16), mean 39.16 ± 12.01	100 (65/35), mean 34.33 ± 10.72	use of the Protocol of Tinnitus and Hyperacusis of HC-FMUSP	according to the research diagnostic criteria for TMDs (RDC/TMD) Axes I and II
Morais & Gil, 2012 [30]	Brazil	cross-sectional	20 (14/6), mean 32.1	n/a	identification of the tinnitus pitch and loudness by means of acuphenometry	checklist of TMD signs and symptoms
Pavaci et al., 2019 [31]	Italy	case-control	130 (55/75), mean 53.1 ± 13.97	100 (46/54), mean 46.16 ± 12.95	hyperacusis questionnaire (HQ) and the tinnitus handicap inventory (THI) in a self-administered way	not specified
Peleg et al., 2022 [16]	Israel	cross-sectional	51 (24/27), 51.26 ± 17.97	n/a	tinnitus for at least 6 months as reported by a patient; confirmed based on physical and an audiometric examination	according to the diagnostic criteria for TMDs (DC/TMD) Axes I and II
Stohler et al., 2019 [32]	the United Kingdom	case-control	109,783 (55,909/53,874), 54.7 ± 15.9	109,783 (55,909/53,874), age-matched	first-time-diagnosed tinnitus	not specified
Tullberg & Ernberg, 2006 [33]	Sweden	cross-sectional	120 (56/64), 47 ± 13	n/a	not specified	presence of TMD symptoms and signs
Vielsmeier et al., 2012 [3]	Germany	cross-sectional	1204 (451/753), 52.97 ± 13.28	n/a	according to Tinnitus Sample Case History Questionnaire	self-reported TMJ problems

Legend: F, female; M, male; n/a, not applicable; TMDs, temporomandibular disorders; TMJ, temporomandibular joint.

**Table 3:** Detailed results of meta-analysis considering pooled prevalence of temporomandibular disorders in tinnitus patients.

Study	Sample Size	Proportion (%)	95% CI	Weight (%)
Attanasio et al., 2015 [26]	55	27.273	16.138 to 40.962	8.97
Cimino et al., 2022 [27]	79	67.089	55.601 to 77.252	9.10
Didier et al., 2023 [1]	47	97.872	88.706 to 99.946	8.90
Edvall et al., 2019 [28]	2482	19.581	18.036 to 21.198	9.40
Hilgenberg et al., 2012 [29]	100	85.000	76.469 to 91.355	9.16
Morais & Gil, 2012 [30]	20	90.000	68.302 to 98.765	8.31
Pavaci et al., 2019 [31]	130	48.462	39.610 to 57.385	9.22
Peleg et al., 2022 [16]	51	27.451	15.893 to 41.745	8.94
Stohler et al., 2019 [32]	109,783	2.501	2.410 to 2.595	9.41
Tullberg & Ernberg, 2006 [33]	120	80.000	71.719 to 86.745	9.20
Vielsmeier et al., 2012 [3]	1204	21.678	19.380 to 24.114	9.39
Total (random effects)	114,071	50.988	33.305 to 68.544	100.00
Egger’s test (publication bias)		Intercept	95% CI	*p*-value
		16.3805	10.1555 to 22.6055	0 = 0.0002

**Table 4:** Detailed results of meta-analysis considering pooled odds ratio of temporomandibular disorders in tinnitus patients.

Study	Tinnitus	Controls	Odds Ratio	95% CI	*p*-Value	Weight (%)
Hilgenberg et al., 2012 [29]	85/100	55/100	4.636	2.359 to 9.112		25.66
Pavaci et al., 2019 [31]	63/130	33/100	1.909	1.112 to 3.277		30.25
Stohler et al., 2019 [32]	2746/109,783	1662/109,783	1.669	1.569 to 1.775		44.09
Total (random effects)	2894/110,013	1750/109,983	2.259	1.334 to 3.827	0.002	100.00
Egger’s test (publication bias)			Intercept	95% CI		
			1.9433	−15.4477 to 19.3343	0.3906	

## Data Availability

No new data were created or analysed in this study.
